# Potential drugs against COVID-19 revealed by gene expression profile, molecular docking and molecular dynamic simulation

**DOI:** 10.2217/fvl-2020-0392

**Published:** 2021-07-20

**Authors:** Claudia Cava, Gloria Bertoli, Isabella Castiglioni

**Affiliations:** 1 ^1^Institute of Molecular Bioimaging & Physiology, National Research Council (IBFM-CNR), Via F. Cervi 93, Segrate-Milan, Milan, 20090, Italy; 2 ^2^Department of Physics “Giuseppe Occhialini”, University of Milan-Bicocca Piazza dell'Ateneo Nuovo, Milan, 20126, Italy

**Keywords:** 3CL main protease, bioinformatics, COVID-19, drug, dynamic simulation, gene expression, interactions, molecular docking, SARS-CoV, therapy

## Abstract

**Aim:** SARS-CoV-2, an emerging betacoronavirus, is the causative agent of COVID-19. Currently, there are few specific and selective antiviral drugs for the treatment and vaccines to prevent contagion. However, their long-term effects can be revealed after several years, and new drugs for COVID-19 should continue to be investigated. **Materials & methods:** In the first step of our study we identified, through a gene expression analysis, several drugs that could act on the biological pathways altered in COVID-19. In the second step, we performed a docking simulation to test the properties of the identified drugs to target SARS-CoV-2. **Results:** The drugs that showed a higher binding affinity are bardoxolone (-8.78 kcal/mol), irinotecan (-8.40 kcal/mol) and pyrotinib (-8.40 kcal/mol). **Conclusion:** We suggested some drugs that could be efficient in treating COVID-19.

A state of global health emergency has been declared by WHO for COVID-19. The causative agent is SARS-CoV-2, which like SARS-CoV and MERS-CoV, belongs to the betacoronavirus genus [1].

The most functionally relevant proteins in SARS-CoV-2 are the spike glycoprotein and 3CL main protease. The spike protein binds to a host cell membrane and allows the entry of the virus into the host cell. It has been described that SARS-CoV entrance is mediated by angiotensin-converting enzyme 2 (ACE2) that is particularly overexpressed by epithelial cells in oral mucosa and intestine [1].

Previous studies have shown that the interaction between the spike glycoprotein and ACE2 has a key role in SARS pathogenesis. Thus, initially, these two proteins have been proposed as possible and promising drug targets. However, anti-viral agents targeting ACE2 and spike glycoprotein did not proceed clinically due to significant side effects [2,3].

Several studies also demonstrated the potential of the 3CL main protease as a promising drug target [4,5]. 3CL main protease is responsible for the regulation of the polyproteins that are translated from the viral RNA [6]. The inhibition of this enzyme could block the replication processes of the virus, which makes it a potential target for drug discovery. Recently x-ray crystal structure of the 3CL main protease was determined providing an excellent basis for structure-based drug discovery [7]. To date, no effective SARS-CoV-3CL inhibitor has been proposed yet to treat COVID-19.

In order to identify new potential targets to block COVID-19 disease, *in silico* drug discovery approaches have been demonstrated to be effective, efficient and cost saving and can follow two main strategies. The first strategy is based on the analysis of gene expression profiles of patients affected by COVID-19, to identify new potential drug targets acting on molecular mechanisms altered by SARS-CoV-2. Several studies based on this approach allowed researchers to discovery candidate drugs for different viruses, such as SARS-CoV-2, MERS-CoV, Ebola and Zika virus [8–11]. The second strategy is to test existing drugs with a virtual screening approach based on the study of the 3D structures of the target protein. This approach, called molecular docking, can predict how drug candidates bind to a receptor of known 3D structure [12], generating a score (binding energy value) that defines the binding affinities between ligand and target protein. There have been successful applications that demonstrated how molecular docking is becoming a powerful tool in the discovery of drug candidates [13,14]. However, most of these studies tested the drugs without considering the effect of drugs on genes deregulated as effect of diseases [1,15]. Indeed, the novelty of the present study is the combination of gene expression analyses with molecular docking and dynamic simulation. In our study, the selection of a gene as therapeutic target was performed via a gene expression analysis and, since the optimal target was identified, the known interactions between drugs and targets were investigated through the study of their binding energies using molecular docking and simulation studies. To our knowledge there are no studies in the literature that integrate these two techniques in COVID-19. Currently, published works screened a library of drugs or molecules against SARS-CoV-2, but without a prior selection based on drugs that could regulate genes altered in COVID-19 as obtained from a gene expression analysis [16–18]. Other studies utilized quantitative structure–activity relationship (such as [19]) or binding energies [20] without considering COVID-19 affected cascades of genes. The gene expression profiles altered by COVID-19 supported the docking results and offered a new perspective for subsequent studies.

Although docking programs are fast and easy to use, they are affected by too low accuracy and stability. Indeed, several studies reported that binding free energies based on molecular dynamic simulations are much more reliable [21].

In this study, we proposed a bioinformatic approach based on molecular docking, molecular dynamic simulations and gene expression profiles of COVID-19 to be used *in silico* to test existing drugs as therapies against COVID-19.

## Materials & methods

**Figure 1.**
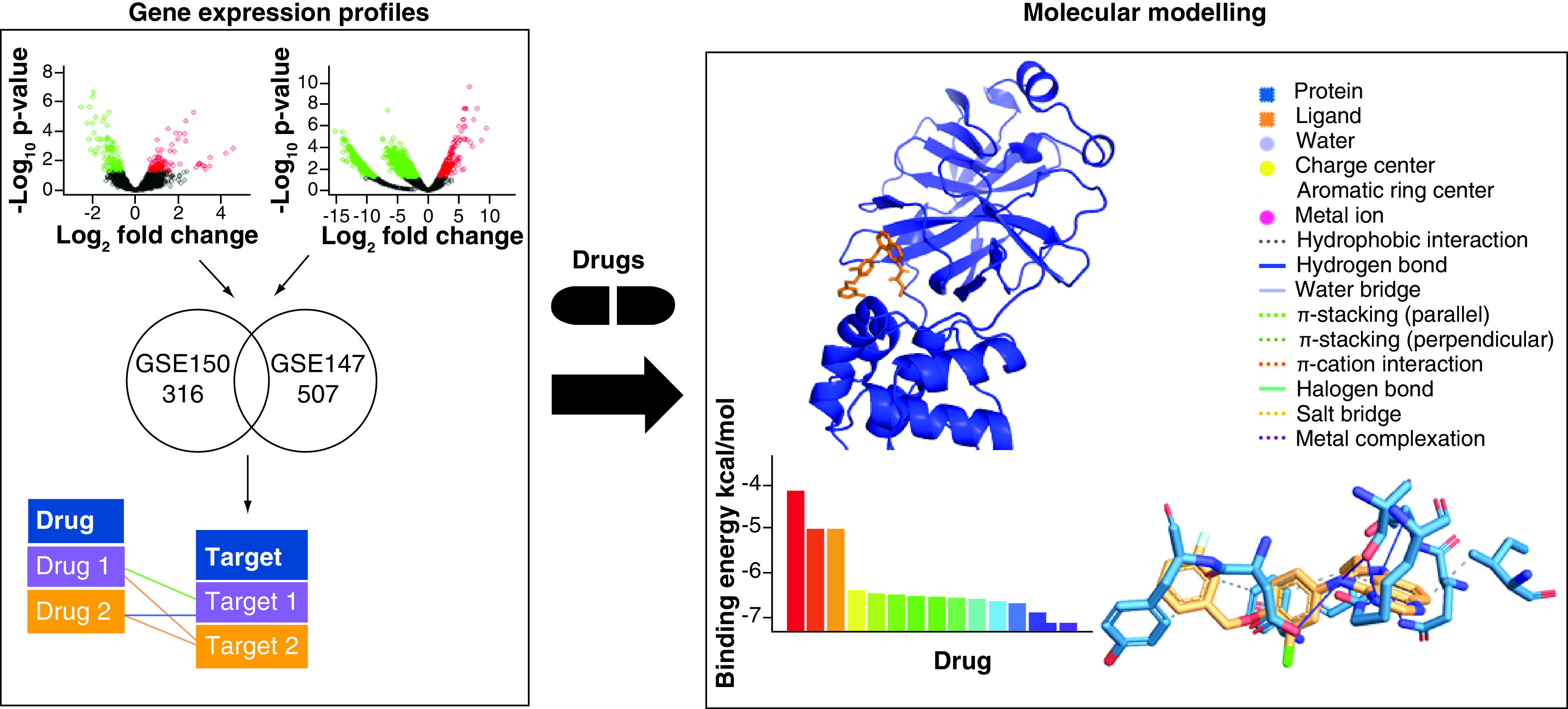
Workflow of the proposed approach. A computational pipeline based on gene expression data analysis, molecular docking and molecular dynamic simulation was adopted for *in silico* drug discovery.

In the first step of our study we identified, through a gene expression analysis, several drugs that could act on the biological pathways altered by COVID-19. In particular, we selected differentially expressed genes between COVID-19 and healthy samples, obtained from two published datasets. In the second step, we performed a docking simulation in order to test the properties of these drugs to target the 3CL main protease of SARS-CoV-2. Figure 1 shows the workflow of the proposed approach.

### Gene expression data

We analyzed the gene expression levels of COVID-19 positive and negative patients from two Gene Expression Omnibus (GEO) datasets: GSE150316 and GSE147507.

Specifically, we selected five negative-control samples of lung tissues and 16 COVID-19-positive lung samples from GSE150316; and two lung biopsies from COVID-19-negative controls and two lung biopsies from post-mortem COVID-19-positive patients from GSE147507.

We defined differentially expressed genes between COVID-19-negative and -positive samples if the genes have an absolute value of log fold change >0.5 and adjusted p-value < 0.05. Pre-processing and differential expression analyses were performed with the Bioconductor package TCGAbiolinks [22].

### Drug interactions & overrepresentation analysis

Matador and DGIdb databases were used to identify the interactions between the drug and protein [23,24].

We performed a Fisher's test to verify if there is an overrepresentation of drug targets in the list of differentially expressed genes. We considered drugs acting on the differentially expressed genes if FDR <0.05. P-values were adjusted with Benjamini–Hochberg procedure for multiple testing correction [25].

### Molecular modeling

We performed molecular docking studies in order to identify potential agents with antiviral properties against COVID-19. Molecular modeling was performed with AutodockTools 1.5.6 [26].

Drugs considered in the overrepresentation analysis were selected and PubChem database was used to extract 3D structure of the selected drugs [27]. Discovery studio was used to prepare ligand (drugs) and generate Protein Data Bank (PDB) format [28]. PDB format is a standard representation for molecular structure data originated from x-ray diffraction.

The crystal structure of COVID-19 main protease in complex with an inhibitor N3 was extracted from PDB (IDs: 6LU7). PDB ID 6LU7 consists of two chains: chain A and chain C. The chain C, representing the complex-bound inhibitor to the protein receptor molecule, was removed. During the pre-processing step of protein polar hydrogen atoms were added and water molecules were removed.

Molecular docking identifies the amino acids that interact between the selected protein and drugs. Minimum energy binding of the ligand with the receptor was considered. Binding energy is described as a decreasing of the overall energy of the complex when a drug is associated with a target protein. Binding energy also defines the ligand binding affinity.

Protein–ligand interaction profiler was used to visualize the binding interactions of the selected drugs with 3D model of protease of COVID-19 [29].

### Molecular dynamics

The investigated main protease of SARS-CoV-2 and screened molecules resulting from molecular docking were subjected to molecular dynamic simulation. The results obtained through the molecular docking were extended, as molecular dynamics are considered a more solid analysis for molecular study of ligand recognition.

Molecular dynamic simulations of the main protease of SARS-CoV-2 with screened molecules were performed using BIOVIA Discovery Studio Client (Dassault Systémes, Vélizy-Villacoublay, France) [30]. Molecular dynamics simulations were carried out using the standard dynamics cascade protocol in four steps: minimization, heating, equilibration run, and production run. We set simulation time (ps) parameters to 20 and 200 in equilibration and production step, respectively. For the other parameters, we used the default values.

Minimization of the 3D structures to get the most stable confirmations of the complexes was performed using the default algorithms: the steepest descent algorithm and the adopted basis NR algorithm.

After the minimization step, all the complexes were subjected to gradual heating using the default values.

In the equilibration phase, the system was stabilized at a target temperature, as the energy has to be distributed appropriately among all systems.

Furthermore, complexes were subjected to the production run at constant temperature, using constant-volume ensemble (NVT). The results of this step are stored in the simulation trajectory.

The average binding energy was calculated for equilibrated molecular dynamic trajectory. Trajectory file contains 100 conformations. The binding energy was calculated by:

(Eq. 1) $$\Delta {\text{G}}_{\text{bind}}=\Delta {\text{G}}_{\text{complex}}-\Delta {\text{G}}_{\text{protein}}-\Delta {\text{G}}_{\text{ligand}}$$

where ΔG_complex_, ΔG_protein_ and ΔG_ligand_ are the total free energy of protein–ligand complex, protein and ligand in solvent, respectively.

### Pharmacokinetics parameters

Lipinski's rule is an algorithm consisting of a set of rules to be considered for the design and development of a drug [30]. It is based on four criteria which must be respected: molecular mass should be less than 500 daltons; the drug should not have more than five hydrogen bond donors in its skeleton; the drug should not have more than five hydrogen bond acceptors in its skeleton; and the fat solubility of the molecule expressed by the partition coefficient must be less than 5. In summary, Lipinski's rule considers two main concepts: absorption (drug candidate should be better absorbed if small in size) and permeation (drug candidate should better pass through the cell membranes if it is not too hydrophilic). We used the SwissADME tool to verify if the selected drugs respect the above-reported criteria [30]. We used ChEBI database to download mol file for the selected drugs [31]. Mol file, a commonly used chemical structure file format is used as input for SwissADME tool.

## Results

### Gene expression analysis

We obtained 198 differentially expressed genes from GSE150316 and 2670 genes from GSE147507. We found 47 genes that are differentially expressed in both datasets.

We then identified 38 drugs (FDR <0.0211) that have an overrepresentation of gene targets in the list of 47 differentially expressed genes. Some of these drugs showed an antiviral property against different viral diseases such as bardoxolone, irinotecan, olaparib, inosine and anthraquinone. The mechanism of action of these drugs are different such as inhibition of protein kinase, inhibition of epidermal growth factor receptor and induction of apoptosis.

27 out of 38 drugs were tested for docking analysis and are depicted in the Table 1. The 3D structure of Hemay-022, lapuleucel-T, T-DM1, ertumaxomab, seribantumab, onartuzumab, depatuxizumab mafodotin, MDX-210, MM-302, trodusquemine and margetuximab are not available from PubCHEM.

**Table 1.** Drugs enriched with differentially-expressed gene targets.

	Drug	Structure	Target gene	Mechanism
1	AZD-8055		*ERBB2*, *MAPKAP1*	mTOR inhibitor
2	Olaparib		*PARP1*, *ERBB2*	Inhibitor of the nuclear enzyme poly(ADP-ribose) polymerase
3	Irinotecan		*ERBB2*, *TOP1MT*	Inhibitor of topoisomerase I activity
4	Niacinamide		*PARP1*	Inhibitor of poly(ADP-ribose) polymerases
5	Heptanoate		*PARP1*	Plant metabolite
6	Iniparib		*PARP1*	Potential cytotoxic activity
7	Tyrphostin AG 879		*ERBB2*	Inhibitor of tyrosine kinase
8	Irbinitinib		*ERBB2*	Inhibitor of the human epidermal growth factor receptor tyrosine kinase ErbB-2
9	Inosine		*PARP1*	Purine nucleoside
10	Anthraquinone		*ERBB2*	Polycyclic aromatic hydrocarbon derived from anthracene or phthalic anhydride
11	Doxorubicin		*GADD45A*, *ERBB2*	Inhibitor of protein synthesis
12	Omtriptolide Sodium		*NFKB1*	Inhibitor of topoisomerase II
13	Topotecan Hydrochloride		*TOP1MT*	Inhibitor of topoisomerase
14	TAK-285		*ERBB2*	Inhibitor of epidermal growth factor receptor
15	Mubritinib		*ERBB2*	Protein kinase inhibitor
16	MP-412		*ERBB2*	Protein kinase inhibitor
17	Canertinib		*ERBB2*	Protein kinase inhibitor
18	INSM-18		*ERBB2*	Inhibitor of two receptor tyrosine kinases, the insulin-like growth factor receptor and the c-erbB2/HER2/neu receptor
19	S-222611		*ERBB2*	Protein kinase inhibitor
20	Allitinib		*ERBB2*	Inhibitor of the epidermal growth factor receptor 1 and human epidermal receptor 2
21	7-Ethyl-10-Hydroxy-Camptothecin		*TOP1MT*	Apoptosis inducer
22	Talazoparib Tosylate		*PARP1*	Inhibitor of the nuclear enzyme poly(ADP-ribose) polymerase
23	Varlitinib		*ERBB2*	Inhibitor of the epidermal growth factor receptor 1 and human epidermal receptor 2
24	Falnidamol		*ERBB2*	Epidermal growth factor receptor inhibitor
25	Pyrotinib		*ERBB2*	Inhibitor of the epidermal growth factor receptor 1 and human epidermal receptor 2
26	CP-724714		*ERBB2*	Inhibitor of human epidermal receptor 2
27	Bardoxolone		*NFKB1*	Apoptosis inducer

### Docking studies

In order to identify potential drugs for treating COVID-19 patients, a molecular docking analysis was performed on the 27 drugs revealed by the differential expression analysis.

The results from the SARS-CoV-2 3CL main protease are reported in Table 2. 12 out of 27 drugs obtained a binding energy less than -7 kcal/mol (AZD-8055, olaparib, irinotecan, tyrphostin AG 879, topotecan hydrochloride, MP-412, S-222611, allitinib, 7-ethyl-10-hydroxy-camptothecin, falnidamol, pyrotinib and bardoxolone). The study revealed no violation of Lipinski's rule for these drugs. Tyrphostin AG 879, MP-412, S-222611, allitinib, pyrotinib, bardoxolone are not available in ChEBI.

**Table 2.** Docking study results for SARS-CoV-2 3CL main protease.

Drug	Binding energy (kcal/mol)
AZD-8055	-7.5
Olaparib	-7.39
Irinotecan	-8.4
Niacinamide	-4.43
Heptanoate	-4.19
Iniparib	-6.28
Tyrphostin AG 879	-7.67
Irbinitinib	-6.6
Inosine	-4.58
Anthraquinone	-6.2
Doxorubicin	-6.14
Omtriptolide sodium	-6.62
Topotecan hydrochloride	-7.4
TAK-285	-5.43
Mubritinib	-6.51
MP-412	-7.93
Canertinib	-6.04
INSM-18	-6.05
S-222611	-7.15
Allitinib	-7.1
7-ethyl-10-hydroxy-camptothecin	-7.07
Talazoparib tosylate	-6.43
Varlitinib	-6.2
Falnidamol	-7.64
Pyrotinib	-8.4
CP-724714	-6.43
Bardoxolone	-8.78

**Figure 2.**
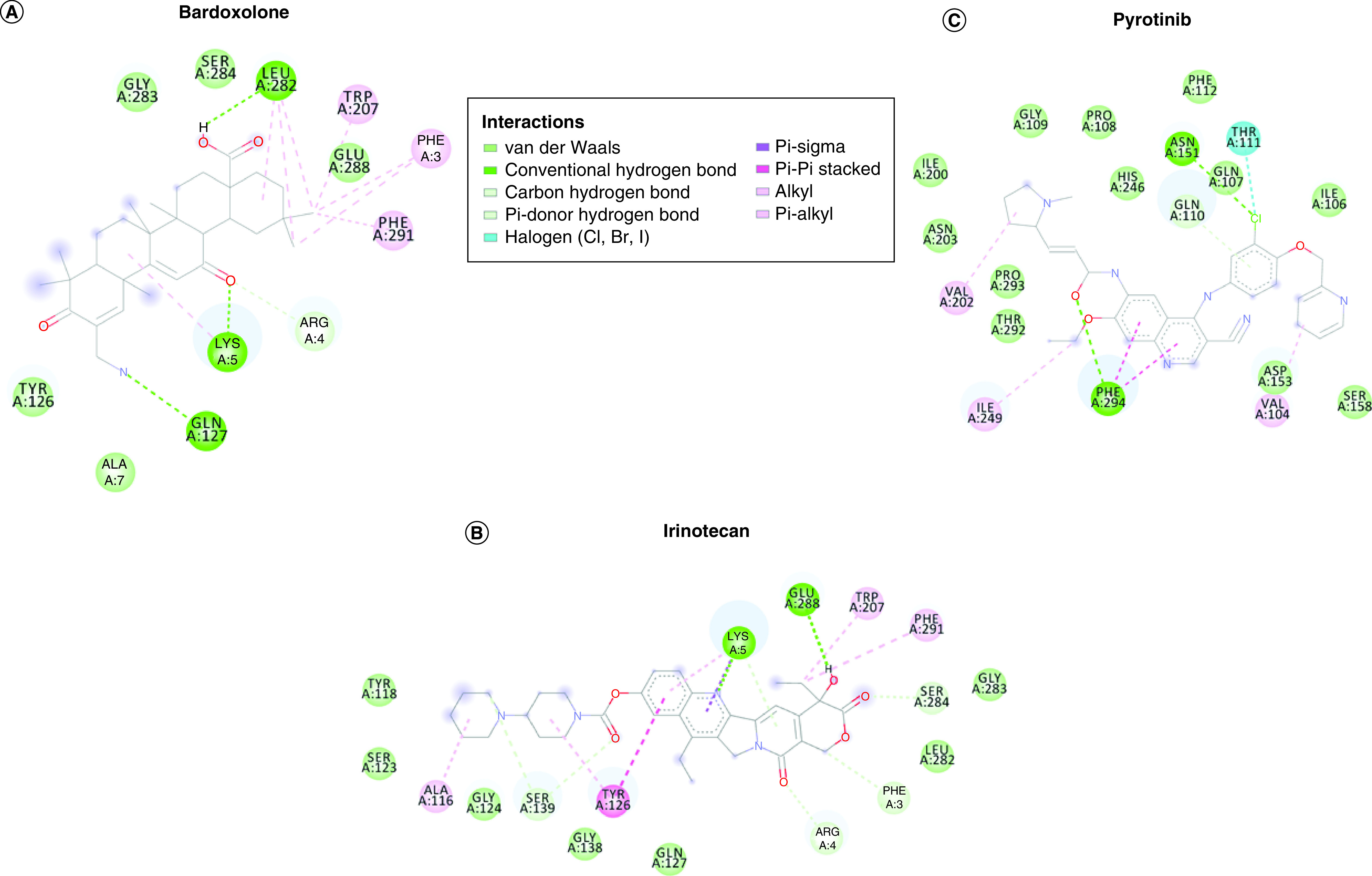
Interaction diagram of three drugs (bardoxolone, irinotecan and pyrotinib) with the main protease of SARS-CoV-2. The figure shows detailed information about protein residues, involved amino acid and type of interactions.

The best binding energy values were obtained for bardoxolone (-8.78 kcal/mol), irinotecan (-8.40 kcal/mol) and pyrotinib (-8.40 kcal/mol). Figure 2 shows the protein–ligand complex for bardoxolone, irinotecan and pyrotinib.

Bardoxolone has four types of interactions with protein residues: van der Waals, hydrogen bonds (conventional and carbon hydrogen bonds), alkyl and pi–alkyl interactions. In particular, the residues LEU282 and LYS5 form alkyl interactions with aromatic ring of the molecule. Moreover, the residue GLN127 interacts with a hydrogen bond with the cyano group of the molecule.

Irinotecan interacts with protein structure through van der Waals, hydrogen bonds, alkyl, pi-alkyl interactions, pi-sigma and pi-pi stacked. The residue TYR126 forms pi-pi stacked and alkyl interactions with aromatic ring of the molecule. Hydroxyl group interacts with GLU288.

Pyrotinib interacts with protein structure through van der Waals, hydrogen bonds, halogen, pi-pi stacked and alkyl interactions. In particular, chloro of the ligand interacts with the residue THR111 through halogen interaction and with the residue ASN151 through hydrogen interaction. PHE294 forms pi-pi stacked interactions with the aromatic rings of the molecule.

Supplementary File 1 shows the binding site predictions for each of the 27 drugs against SARS-CoV-2 3CL main protease.

### Molecular dynamic simulation

COVID-19 main protease (PDB IDs: 6LU7) in complex with the top three (bardoxolone, irinotecan and pyrotinib) docked ligands have been selected to compute dynamic properties through molecular dynamic simulations. The calculated binding energies are presented in Table 3. All three ligands obtained negative binding energies. Pyrotinib exhibited the lowest binding energy (-291 kcal/mol). Irinotecan achieved a binding energy of -240 kcal/mol and bardoxolone of -160 kcal/mol.

**Table 3.** Binding energies based on molecular dynamic simulations for three drugs: bardoxolone, irinotecan and pyrotinib.

Ligands	ΔG kcal/mol
Bardoxolone	-160,887 ± 5,877
Irinotecan	-240,473 ± 12,521
Pyrotinib	-291,571 ± 15,627

## Discussion

Previous studies demonstrated the emergent ability of mesenchymal stem cells (MSCs), and adipose-derived mesenchymal stem cells (AD-MSCs) as antiviral agents in patients with COVID-19 having properties of immunomodulation, regeneration and repair and antimicrobial [32–35]. MSC-based treatments have been reported in several autoimmune disorders successfully, but they have not still been led to a standard therapy [36]. Indeed, the US FDA did not approve MSCs for the treatment of COVID-19 because of limited data studied [36].

To date, FDA has approved five therapies for COVID-19: remdesivir, sotrovimab, combination of bamlanivimab and etesevimab, combination of casirivimab and imdevimab and COVID-19 convalescent plasma [37].

Remdesivir was one of the first antiviral drugs used in COVID-19 treatment and was authorised in over 50 countries [36]. It is an adenosine nucleotide analogue and inhibits the viral RNA-dependent RNA polymerase blocking the virus replication [38].

Sotrovimab is a recombinant human monoclonal antibody that binds to spike protein of SARS-CoV-2 and acts by avoiding the virus' entry into human cells [39].

Bamlanivimab and etesevimab, monoclonal antibodies, were approved by FDA to be administered together for the treatment of COVID-19. They are specifically directed against different but overlapping sites of the spike protein of SARS-CoV-2 stopping its binding to the human ACE2 receptor [40]. Similarly, the combination of two recombinant human monoclonal antibodies, casirivimab, and imdevimab, was reported for use in the treatment of COVID-19 [41]. Convalescent plasma (CP), obtained from plasma derived from healthy donors recovered from COVID-19, contains virus-specific antibodies that could provide passive immunity in infected patients [42].

Despite the current standard therapy for COVID-19, new drugs should continue to be investigated as, the long-term effects of drugs and vaccines will be revealed in several years. In addition, recent studies demonstrated that a minimal variation in the sequence of SARS-CoV-2 could lead to modifications in the viral proteins making the vaccines or drugs ineffective [43].

To date, no mutations have been identified on 3CL main protease rendering it an appealing target for novel antiviral drugs [44]. Indeed, another innovative aspect of our computational approach is the focus on 3CL main protease that could help in identifying its inhibitors [45].

In this study, we proposed a novel computational approach integrating gene expression analysis and molecular docking to test *in silico* existing drugs against 3CL main protease of SARS-CoV-2.

In the first step of the study, we analyzed gene expression profiles of COVID-19 patients from two published GEO datasets. We identified 47 common differentially-expressed genes in COVID-19 patients compared with healthy subjects, and we verified the overrepresentation of these genes in the list of drug targets. We obtained 38 drugs as potential promising treatment for COVID-19 patients.

As the 3D structure for several drugs was not available, 27 out of 38 drugs were tested for docking analysis. Docking results suggested three potential protease inhibitors namely bardoxolone (binding energy: -8.78 kcal/mol), irinotecan (binding energy: -8.40 kcal/mol) and pyrotinib (binding energy: -8.40 kcal/mol). However, the best binding energy was obtained by bardoxolone. Based on differential expression and overrepresentation analysis, bardoxolone appears to be the best drug for treating COVID-19 patients. However, as 12 out of 27 drugs obtained a binding energy less than -7 kcal/mol (AZD-8055, olaparib, irinotecan, tyrphostin AG 879, topotecan hydrochloride, MP-412, S-222611, allitinib, 7-ethyl-10-hydroxy-camptothecin, falnidamol, pyrotinib and bardoxolone), these drugs can all be considered potential protease inhibitor ligands.

Bardoxolone was suggested as drug with novel potent antiviral properties against hepatitis B and C viruses in human hepatocyte cell culture systems and herpesvirus [46,47]. Bardoxolone could act as an agonist of activator of NRF2 impairing viral replication [46,47]. NRF2 induces HO-1 that was shown to suppress genome replication in hepatitis B and C. High *NRF2* activity suggests the role of *NRF2* as cellular defence against the progression of the infection. Indeed, several studies reported that the cells with a high level of *NRF2* are less likely to progress the infection. *NRF2* activity was also associated with a decreasing virus production in rotavirus [48].

Irinotecan (CPT-11) is a semisynthetic plant alkaloid derived from camptothecin and acts as an anticancer due to its ability to inhibit topoisomerase I during S phase; it is used to treat various cancers (i.e. colorectal cancer, pancreatic cancer, etc.), but its side effects limits its use. Nevertheless, it has been demonstrated in *in vitro* experiments to be effective in blocking herpes simplex virus-1 replication and lytic oncolysis [49]. Several nanoparticle- or lipid-based formulations have been developed to be used in different viral infectious disease, such as hepatitis A or influenza [50] carriers it is necessary to control the possible reactivation of the virus to avoid the development of impaired liver function or liver cancer. About 20% or HBV carriers developed reactivation [51]. In these subjects, irinotecan is used as a palliative treatment for advanced colorectal cancer, and its combination with lamivudine that inhibits HBV reverse transcription, could be helpful in controlling the virus reactivation [51].

Pyrotinib (Irene) is an anti-HER2 therapeutic target drug. This drug acts by covalently binding to ATP binding sites of the kinase domain: in this way the drug inhibits the formation of homo/heterodimer and auto-phosphorylation of HER family, blocking the signaling of the downstream pathway (RAS/RAF/MEK/MAPK, PI3K/AKT) and stopping the cells in G1. With its mechanism of action, this drug is effective on metastatic breast cancer [52]. Being an irreversible tyrosine kinase inhibitor, it blocks signal transduction through the erythroblastic leukemia viral oncogene homolog (erbB) receptors. Although no publication is present in the literature regarding the possible use of pyrotinib as antiviral agent, it is possible that this molecule interferes with viral entry by inhibiting tyrosine kinase activity of surface receptor. Indeed, it has been already reported that tyrosine kinase activity of Abl protein is necessary for the viral spike S-induced syncytia formation prior to the hemifusion step during infectious bronchitis virus (IBV) infection [53]. Moreover, the authors demonstrated that Abl inhibitor drugs are able to block IBV infection process.

Another promising drug against COVID-19 patients that achieved a binding energy of -7.39 kcal/mol is olaparib. It is utilized in current clinical use as poly adenosine diphosphate-ribose polymerase (PARP) inhibitors. PARP/ARTD enzymes are involved in a wide range of cellular processes such as antiviral response. PARP inhibitors have been shown to be effective in several models of acute respiratory distress syndrome [54]. Furthermore, a consequence of SARS-CoV-2 infection can be inflammatory response flaring out of control induced by IL-1 and IL-6. PARP inhibitors can reduce the expression of IL-1 and IL-6 decreasing the inflammatory response [55].

In conclusion, we proposed several existing drugs with antiviral activity that, alone or in combination, could be considered as drugs against COVID-19. However, it is vital to better understand the clinicopathological features of COVID-19 to design treatment plans leading to favorable outcomes in SARS-CoV-2 infected patients [56,57].

Although our work is the first study on the integration of gene expression, molecular docking and dynamic simulation in COVID-19 to our knowledge, it presents some limitations. Indeed, further docking and dynamic simulation studies are needed to validate the inhibitory effects of these drugs against COVID-19 using reference molecules as gold standard.

## Conclusion

In this study we have demonstrated that an *in silico* approach, based on integration of gene expression analysis of COVID-19-specific genes and molecular docking of therapeutic drugs on target proteins, revealed a new antiviral use of existing drugs on the basis of the interaction between each drug and specific domains on SARS-CoV-2 viral proteins. Dynamic simulation studies confirmed the promising role of three drugs: bardoxolone, irinotecan and pyrotinib.

**Summary points**

- SARS-CoV-2 3CL main protease as a promising drug target.
- Gene expression profiles investigated the differentially-expressed genes in COVID-19 patients.
- The best drugs were chosen for their known interactions with genes altered by COVID-19.
- Molecular docking and dynamic simulation analyses were applied on the best drugs.
