# Supplementary material for: Potential drugs against COVID-19 revealed by gene expression profile, molecular docking and molecular dynamic simulation
Source: Future Virol. 2021 Jul 20:10.2217/fvl-2020-0392. doi: 10.2217/fvl-2020-0392 (PMC8293696; doi:10.2217/fvl-2020-0392)
Supplement: Supplementary file 1 [file Supplementary_File.pptx]

## Slide 1
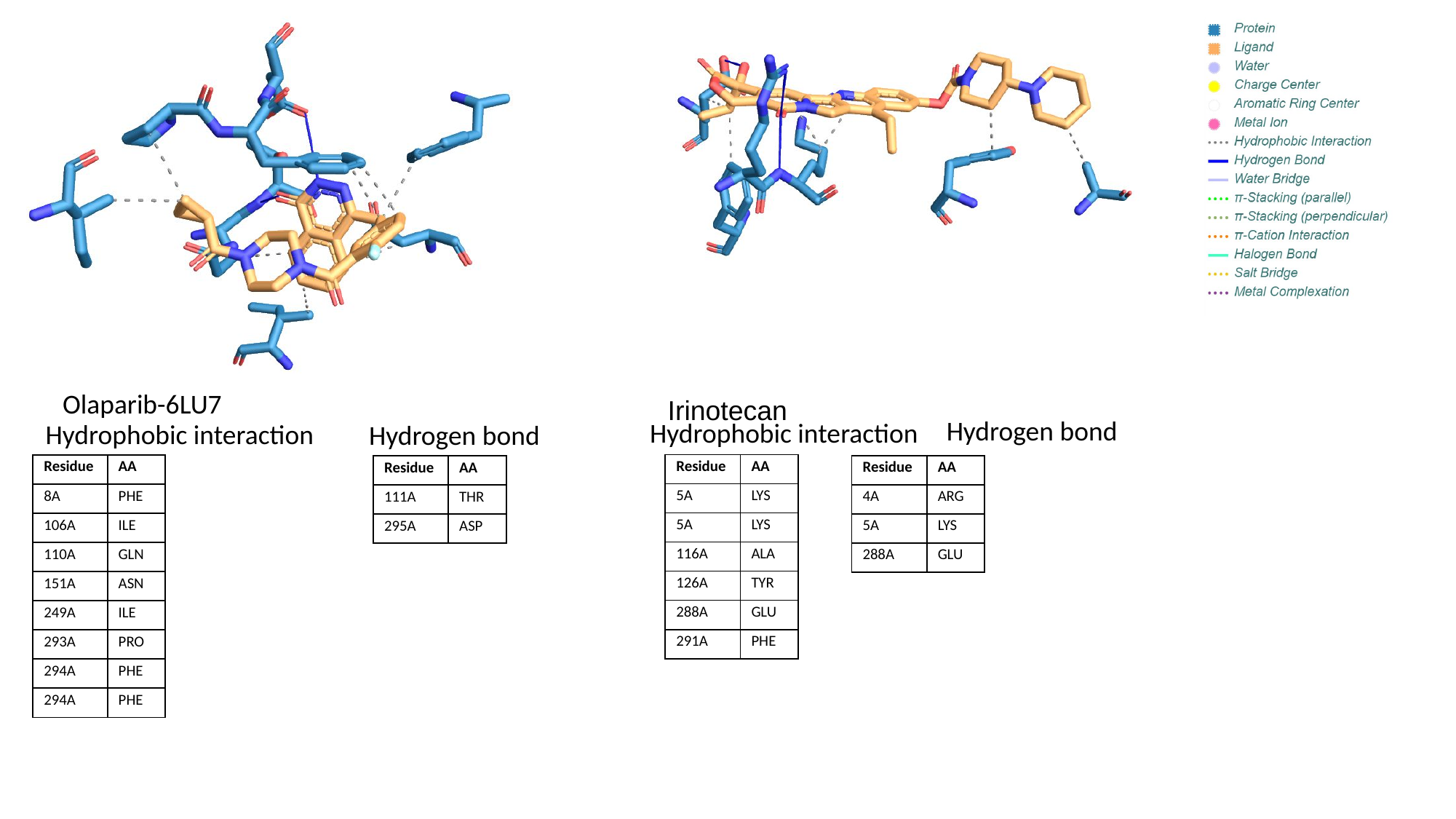

Olaparib-6LU7
Irinotecan
Hydrogen bond
Hydrophobic interaction
Hydrophobic interaction
Hydrogen bond
| Residue | AA |
| --- | --- |
| 5A | LYS |
| 5A | LYS |
| 116A | ALA |
| 126A | TYR |
| 288A | GLU |
| 291A | PHE |
| Residue | AA |
| --- | --- |
| 8A | PHE |
| 106A | ILE |
| 110A | GLN |
| 151A | ASN |
| 249A | ILE |
| 293A | PRO |
| 294A | PHE |
| 294A | PHE |
| Residue | AA |
| --- | --- |
| 4A | ARG |
| 5A | LYS |
| 288A | GLU |
| Residue | AA |
| --- | --- |
| 111A | THR |
| 295A | ASP |

## Slide 2
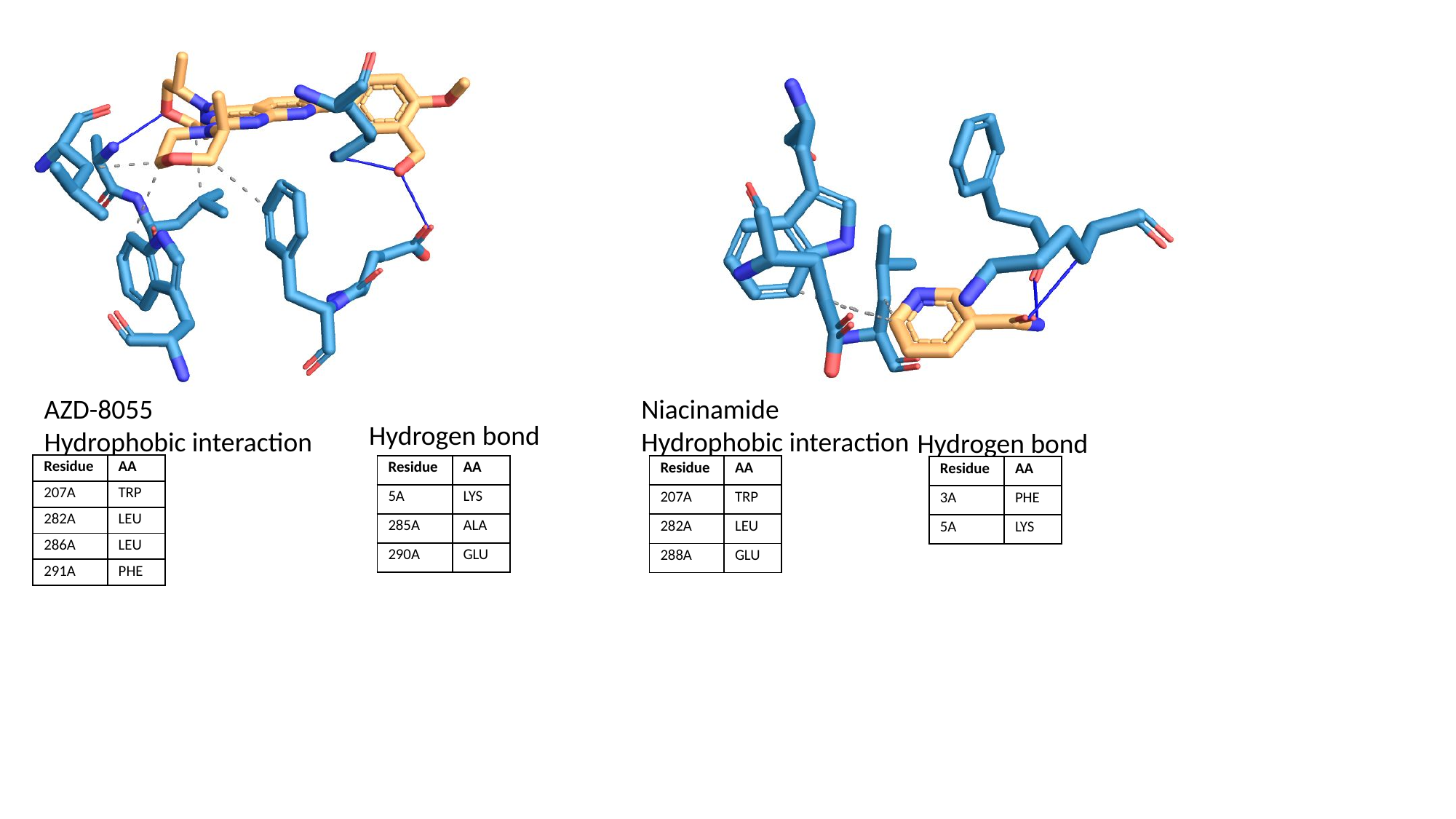

AZD-8055
Hydrophobic interaction
Niacinamide
Hydrophobic interaction
Hydrogen bond
Hydrogen bond
| Residue | AA |
| --- | --- |
| 207A | TRP |
| 282A | LEU |
| 286A | LEU |
| 291A | PHE |
| Residue | AA |
| --- | --- |
| 5A | LYS |
| 285A | ALA |
| 290A | GLU |
| Residue | AA |
| --- | --- |
| 207A | TRP |
| 282A | LEU |
| 288A | GLU |
| Residue | AA |
| --- | --- |
| 3A | PHE |
| 5A | LYS |

## Slide 3
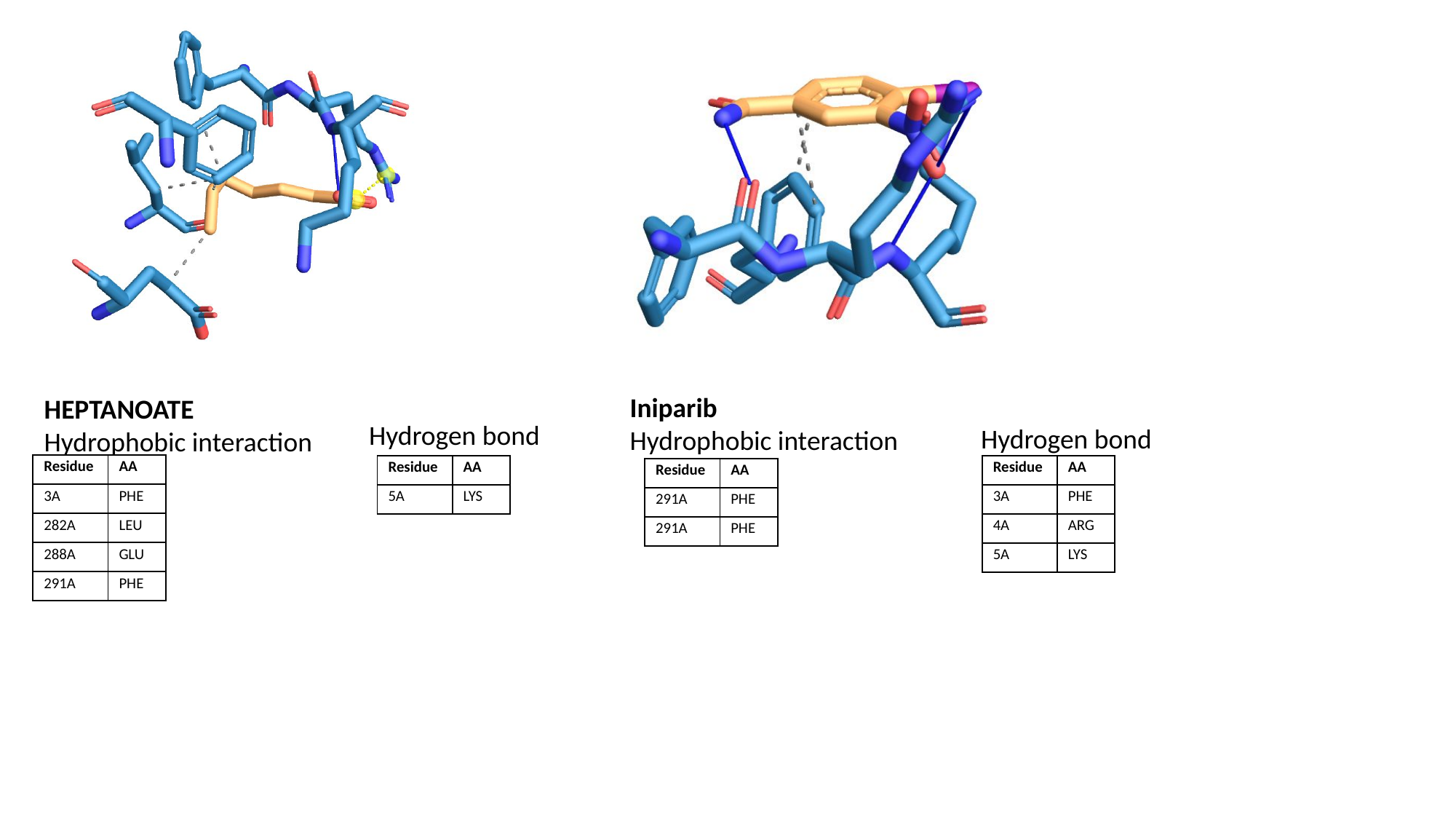

Iniparib
Hydrophobic interaction
HEPTANOATE
Hydrophobic interaction
Hydrogen bond
Hydrogen bond
| Residue | AA |
| --- | --- |
| 3A | PHE |
| 282A | LEU |
| 288A | GLU |
| 291A | PHE |
| Residue | AA |
| --- | --- |
| 5A | LYS |
| Residue | AA |
| --- | --- |
| 3A | PHE |
| 4A | ARG |
| 5A | LYS |
| Residue | AA |
| --- | --- |
| 291A | PHE |
| 291A | PHE |

## Slide 4
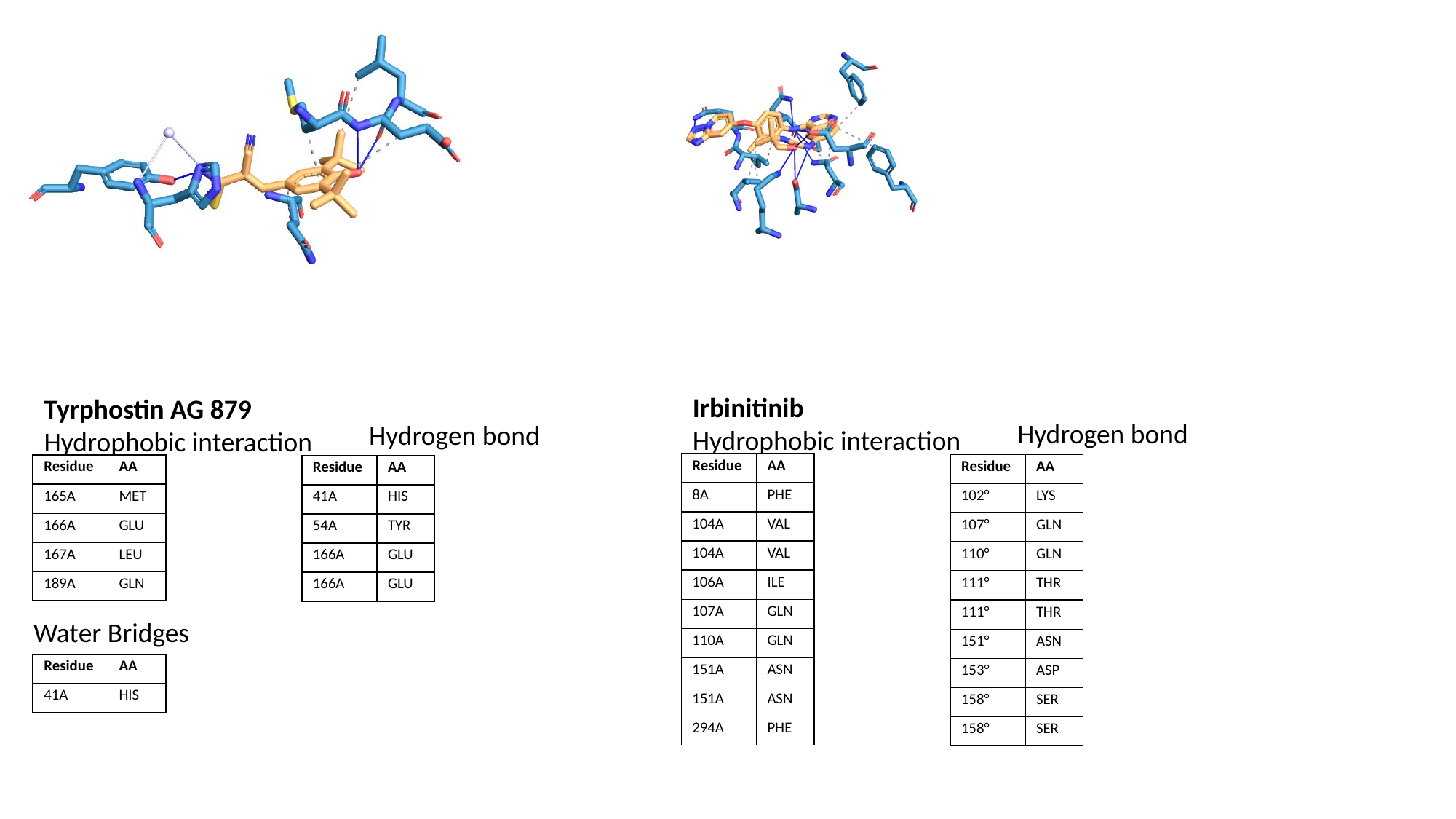

Irbinitinib
Hydrophobic interaction
Tyrphostin AG 879
Hydrophobic interaction
Hydrogen bond
Hydrogen bond
| Residue | AA |
| --- | --- |
| 8A | PHE |
| 104A | VAL |
| 104A | VAL |
| 106A | ILE |
| 107A | GLN |
| 110A | GLN |
| 151A | ASN |
| 151A | ASN |
| 294A | PHE |
| Residue | AA |
| --- | --- |
| 102° | LYS |
| 107° | GLN |
| 110° | GLN |
| 111° | THR |
| 111° | THR |
| 151° | ASN |
| 153° | ASP |
| 158° | SER |
| 158° | SER |
| Residue | AA |
| --- | --- |
| 165A | MET |
| 166A | GLU |
| 167A | LEU |
| 189A | GLN |
| Residue | AA |
| --- | --- |
| 41A | HIS |
| 54A | TYR |
| 166A | GLU |
| 166A | GLU |
Water Bridges
| Residue | AA |
| --- | --- |
| 41A | HIS |

## Slide 5
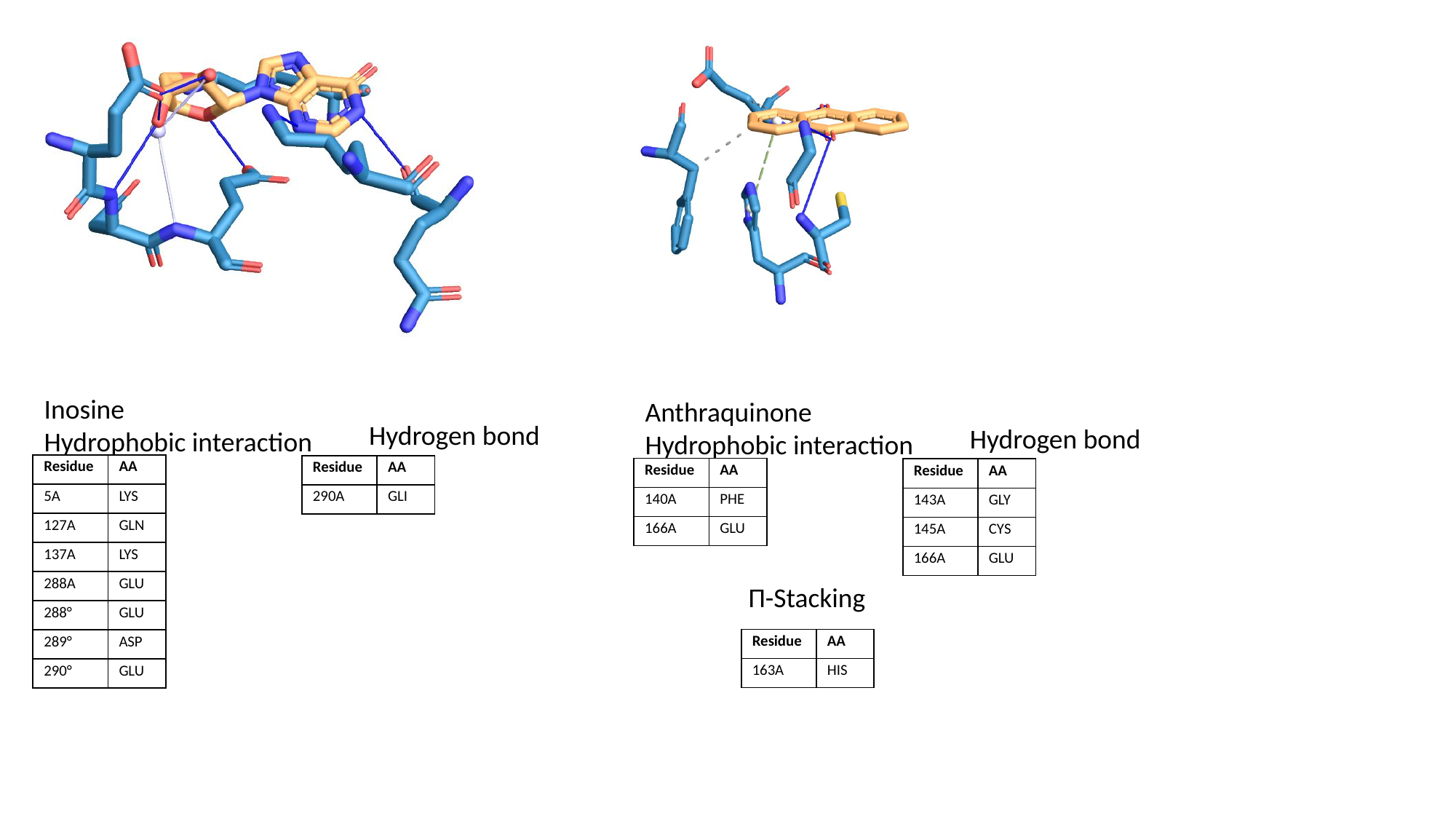

Inosine
Hydrophobic interaction
Anthraquinone
Hydrophobic interaction
Hydrogen bond
Hydrogen bond
| Residue | AA |
| --- | --- |
| 5A | LYS |
| 127A | GLN |
| 137A | LYS |
| 288A | GLU |
| 288° | GLU |
| 289° | ASP |
| 290° | GLU |
| Residue | AA |
| --- | --- |
| 290A | GLI |
| Residue | AA |
| --- | --- |
| 140A | PHE |
| 166A | GLU |
| Residue | AA |
| --- | --- |
| 143A | GLY |
| 145A | CYS |
| 166A | GLU |
Π-Stacking
| Residue | AA |
| --- | --- |
| 163A | HIS |

## Slide 6
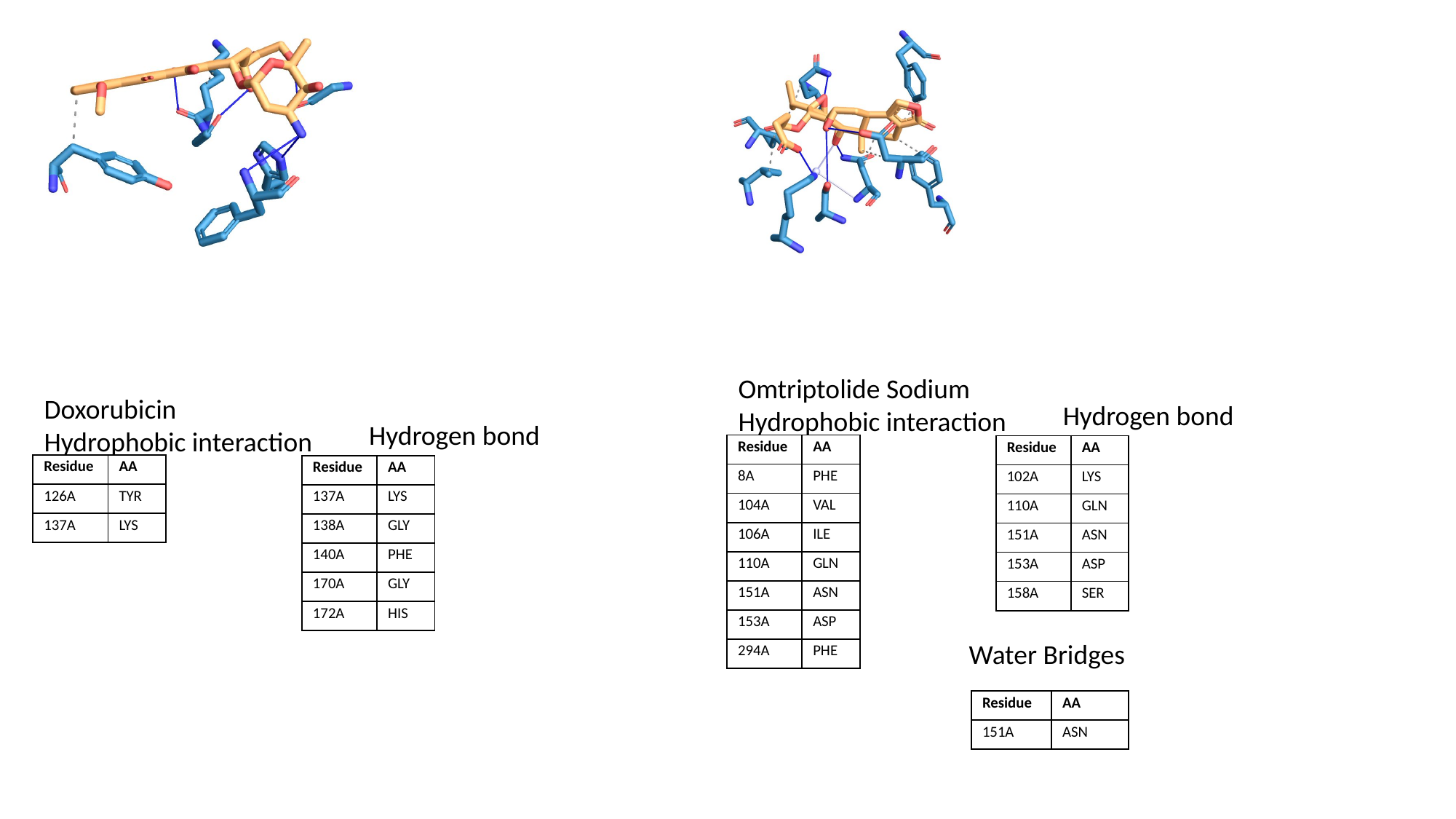

Omtriptolide Sodium
Hydrophobic interaction
Doxorubicin
Hydrophobic interaction
Hydrogen bond
Hydrogen bond
| Residue | AA |
| --- | --- |
| 8A | PHE |
| 104A | VAL |
| 106A | ILE |
| 110A | GLN |
| 151A | ASN |
| 153A | ASP |
| 294A | PHE |
| Residue | AA |
| --- | --- |
| 102A | LYS |
| 110A | GLN |
| 151A | ASN |
| 153A | ASP |
| 158A | SER |
| Residue | AA |
| --- | --- |
| 126A | TYR |
| 137A | LYS |
| Residue | AA |
| --- | --- |
| 137A | LYS |
| 138A | GLY |
| 140A | PHE |
| 170A | GLY |
| 172A | HIS |
Water Bridges
| Residue | AA |
| --- | --- |
| 151A | ASN |

## Slide 7
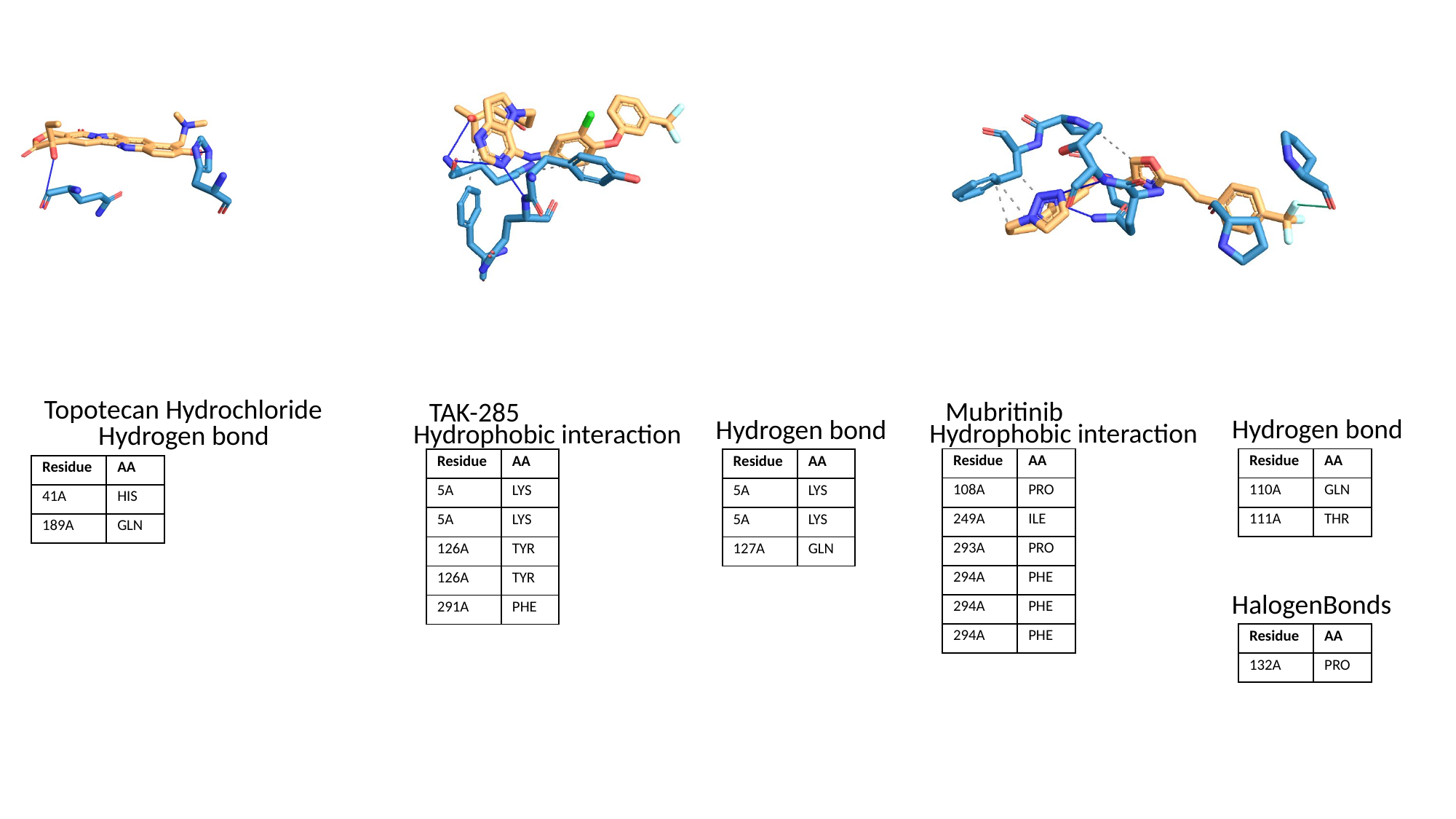

Topotecan Hydrochloride
Mubritinib
TAK-285
Hydrogen bond
Hydrogen bond
Hydrophobic interaction
Hydrophobic interaction
Hydrogen bond
| Residue | AA |
| --- | --- |
| 108A | PRO |
| 249A | ILE |
| 293A | PRO |
| 294A | PHE |
| 294A | PHE |
| 294A | PHE |
| Residue | AA |
| --- | --- |
| 110A | GLN |
| 111A | THR |
| Residue | AA |
| --- | --- |
| 5A | LYS |
| 5A | LYS |
| 126A | TYR |
| 126A | TYR |
| 291A | PHE |
| Residue | AA |
| --- | --- |
| 5A | LYS |
| 5A | LYS |
| 127A | GLN |
| Residue | AA |
| --- | --- |
| 41A | HIS |
| 189A | GLN |
HalogenBonds
| Residue | AA |
| --- | --- |
| 132A | PRO |

## Slide 8
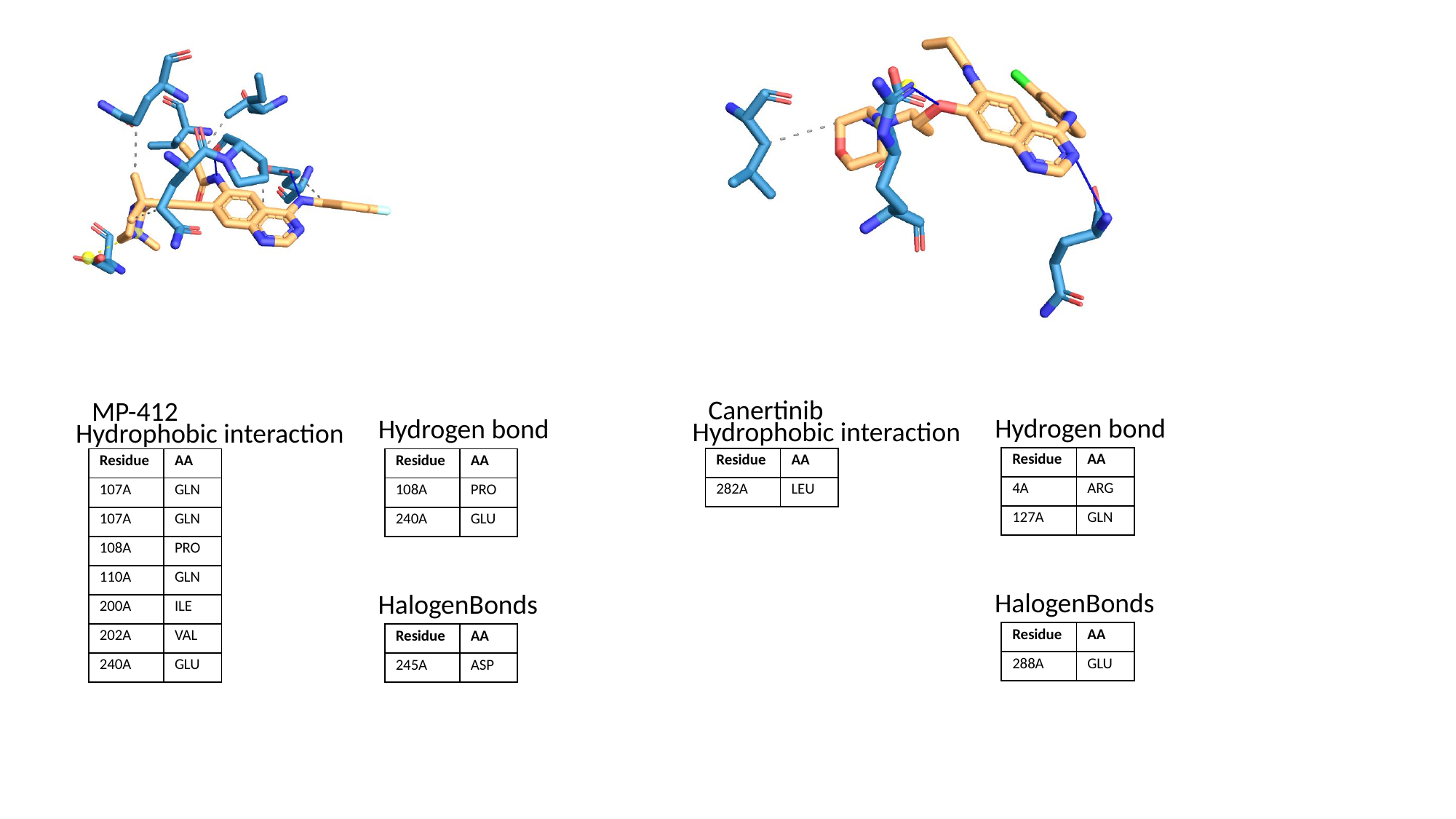

Canertinib
MP-412
Hydrogen bond
Hydrogen bond
Hydrophobic interaction
Hydrophobic interaction
| Residue | AA |
| --- | --- |
| 4A | ARG |
| 127A | GLN |
| Residue | AA |
| --- | --- |
| 282A | LEU |
| Residue | AA |
| --- | --- |
| 107A | GLN |
| 107A | GLN |
| 108A | PRO |
| 110A | GLN |
| 200A | ILE |
| 202A | VAL |
| 240A | GLU |
| Residue | AA |
| --- | --- |
| 108A | PRO |
| 240A | GLU |
HalogenBonds
HalogenBonds
| Residue | AA |
| --- | --- |
| 288A | GLU |
| Residue | AA |
| --- | --- |
| 245A | ASP |

## Slide 9
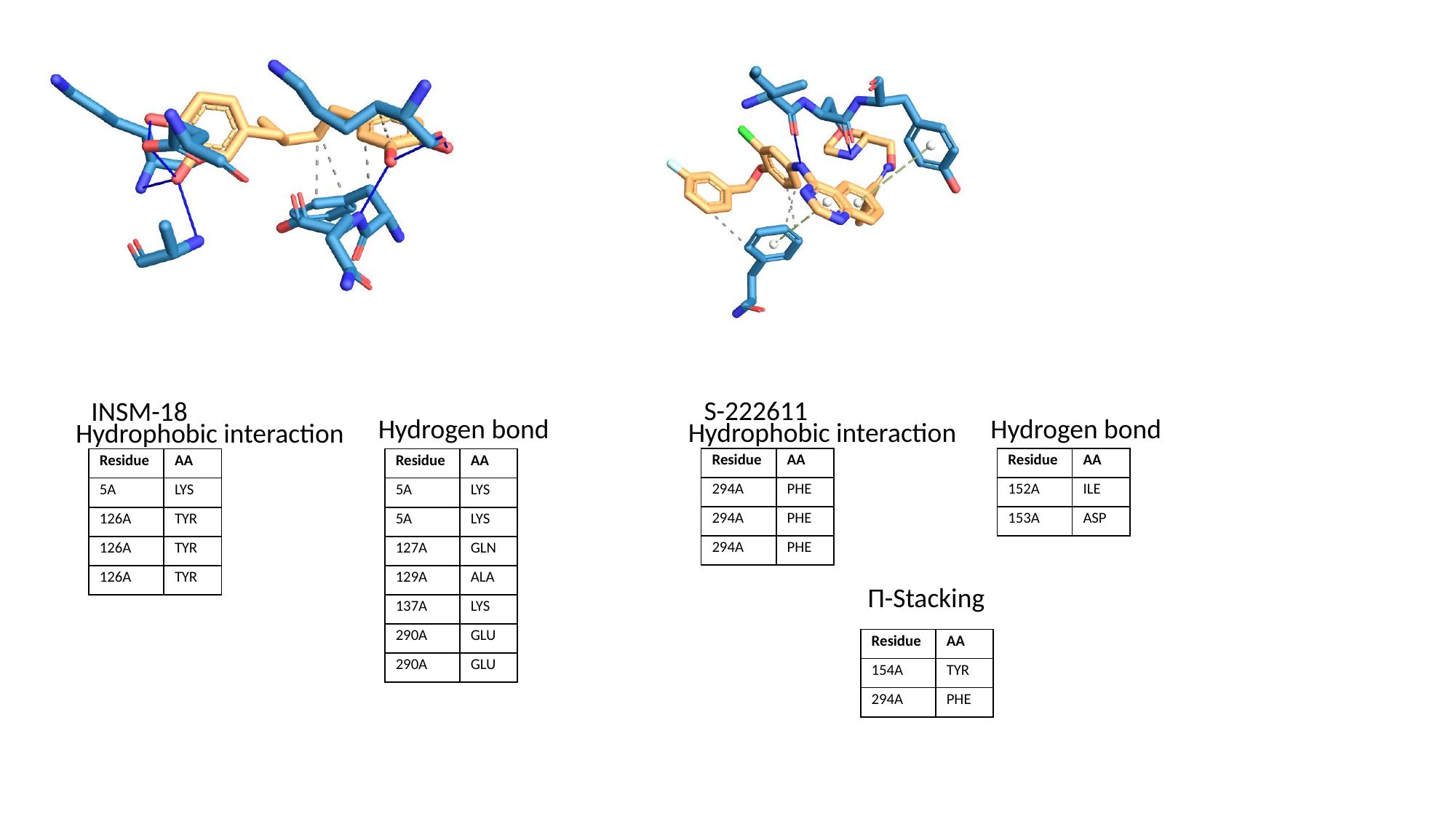

S-222611
INSM-18
Hydrogen bond
Hydrogen bond
Hydrophobic interaction
Hydrophobic interaction
| Residue | AA |
| --- | --- |
| 294A | PHE |
| 294A | PHE |
| 294A | PHE |
| Residue | AA |
| --- | --- |
| 152A | ILE |
| 153A | ASP |
| Residue | AA |
| --- | --- |
| 5A | LYS |
| 126A | TYR |
| 126A | TYR |
| 126A | TYR |
| Residue | AA |
| --- | --- |
| 5A | LYS |
| 5A | LYS |
| 127A | GLN |
| 129A | ALA |
| 137A | LYS |
| 290A | GLU |
| 290A | GLU |
Π-Stacking
| Residue | AA |
| --- | --- |
| 154A | TYR |
| 294A | PHE |

## Slide 10
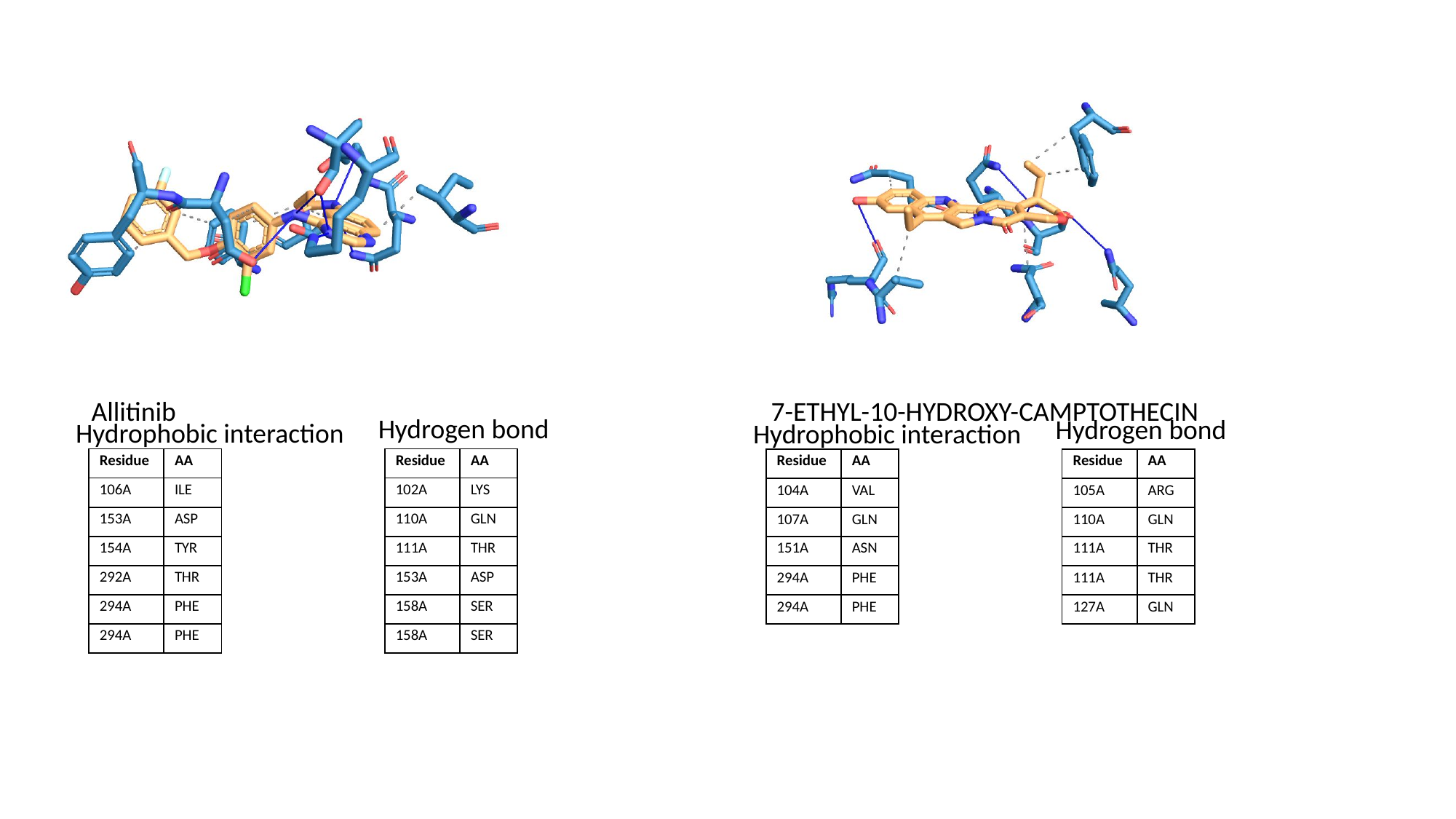

Allitinib
7-ETHYL-10-HYDROXY-CAMPTOTHECIN
Hydrogen bond
Hydrogen bond
Hydrophobic interaction
Hydrophobic interaction
| Residue | AA |
| --- | --- |
| 106A | ILE |
| 153A | ASP |
| 154A | TYR |
| 292A | THR |
| 294A | PHE |
| 294A | PHE |
| Residue | AA |
| --- | --- |
| 102A | LYS |
| 110A | GLN |
| 111A | THR |
| 153A | ASP |
| 158A | SER |
| 158A | SER |
| Residue | AA |
| --- | --- |
| 104A | VAL |
| 107A | GLN |
| 151A | ASN |
| 294A | PHE |
| 294A | PHE |
| Residue | AA |
| --- | --- |
| 105A | ARG |
| 110A | GLN |
| 111A | THR |
| 111A | THR |
| 127A | GLN |

## Slide 11
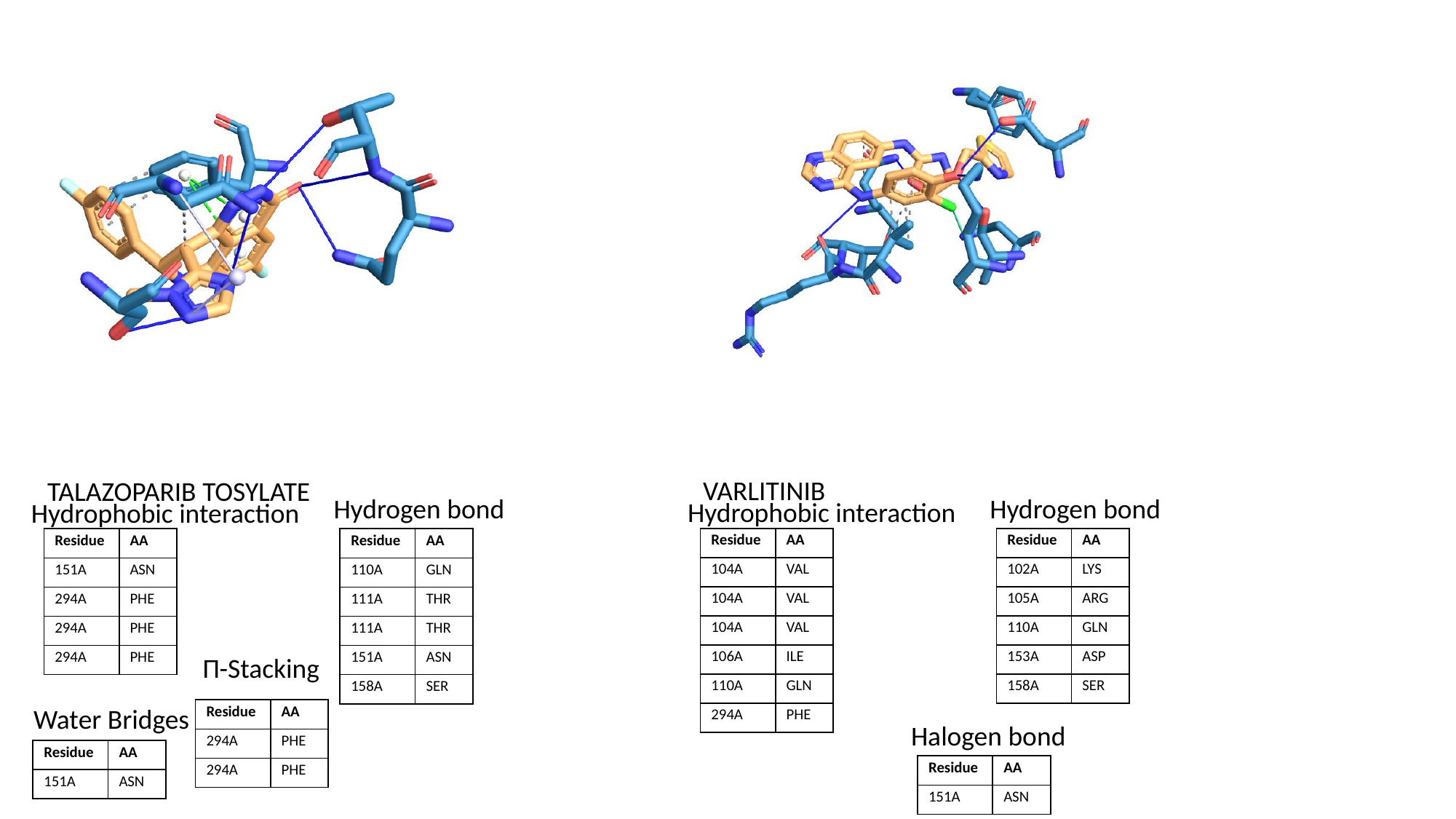

VARLITINIB
TALAZOPARIB TOSYLATE
Hydrogen bond
Hydrogen bond
Hydrophobic interaction
Hydrophobic interaction
| Residue | AA |
| --- | --- |
| 104A | VAL |
| 104A | VAL |
| 104A | VAL |
| 106A | ILE |
| 110A | GLN |
| 294A | PHE |
| Residue | AA |
| --- | --- |
| 102A | LYS |
| 105A | ARG |
| 110A | GLN |
| 153A | ASP |
| 158A | SER |
| Residue | AA |
| --- | --- |
| 151A | ASN |
| 294A | PHE |
| 294A | PHE |
| 294A | PHE |
| Residue | AA |
| --- | --- |
| 110A | GLN |
| 111A | THR |
| 111A | THR |
| 151A | ASN |
| 158A | SER |
Π-Stacking
Water Bridges
| Residue | AA |
| --- | --- |
| 294A | PHE |
| 294A | PHE |
Halogen bond
| Residue | AA |
| --- | --- |
| 151A | ASN |
| Residue | AA |
| --- | --- |
| 151A | ASN |

## Slide 12
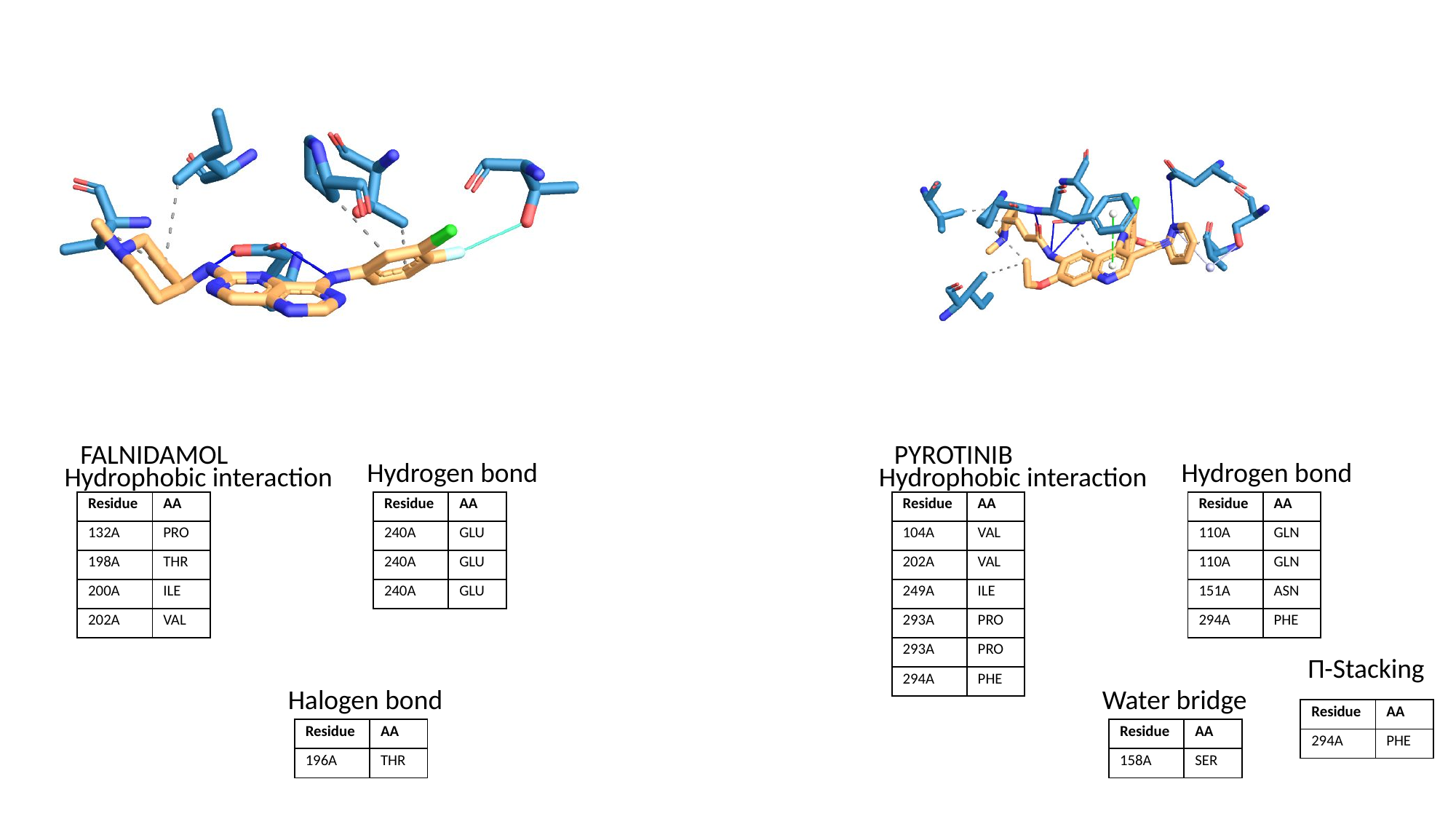

PYROTINIB
FALNIDAMOL
Hydrogen bond
Hydrogen bond
Hydrophobic interaction
Hydrophobic interaction
| Residue | AA |
| --- | --- |
| 104A | VAL |
| 202A | VAL |
| 249A | ILE |
| 293A | PRO |
| 293A | PRO |
| 294A | PHE |
| Residue | AA |
| --- | --- |
| 110A | GLN |
| 110A | GLN |
| 151A | ASN |
| 294A | PHE |
| Residue | AA |
| --- | --- |
| 132A | PRO |
| 198A | THR |
| 200A | ILE |
| 202A | VAL |
| Residue | AA |
| --- | --- |
| 240A | GLU |
| 240A | GLU |
| 240A | GLU |
Π-Stacking
Water bridge
Halogen bond
| Residue | AA |
| --- | --- |
| 294A | PHE |
| Residue | AA |
| --- | --- |
| 158A | SER |
| Residue | AA |
| --- | --- |
| 196A | THR |

## Slide 13
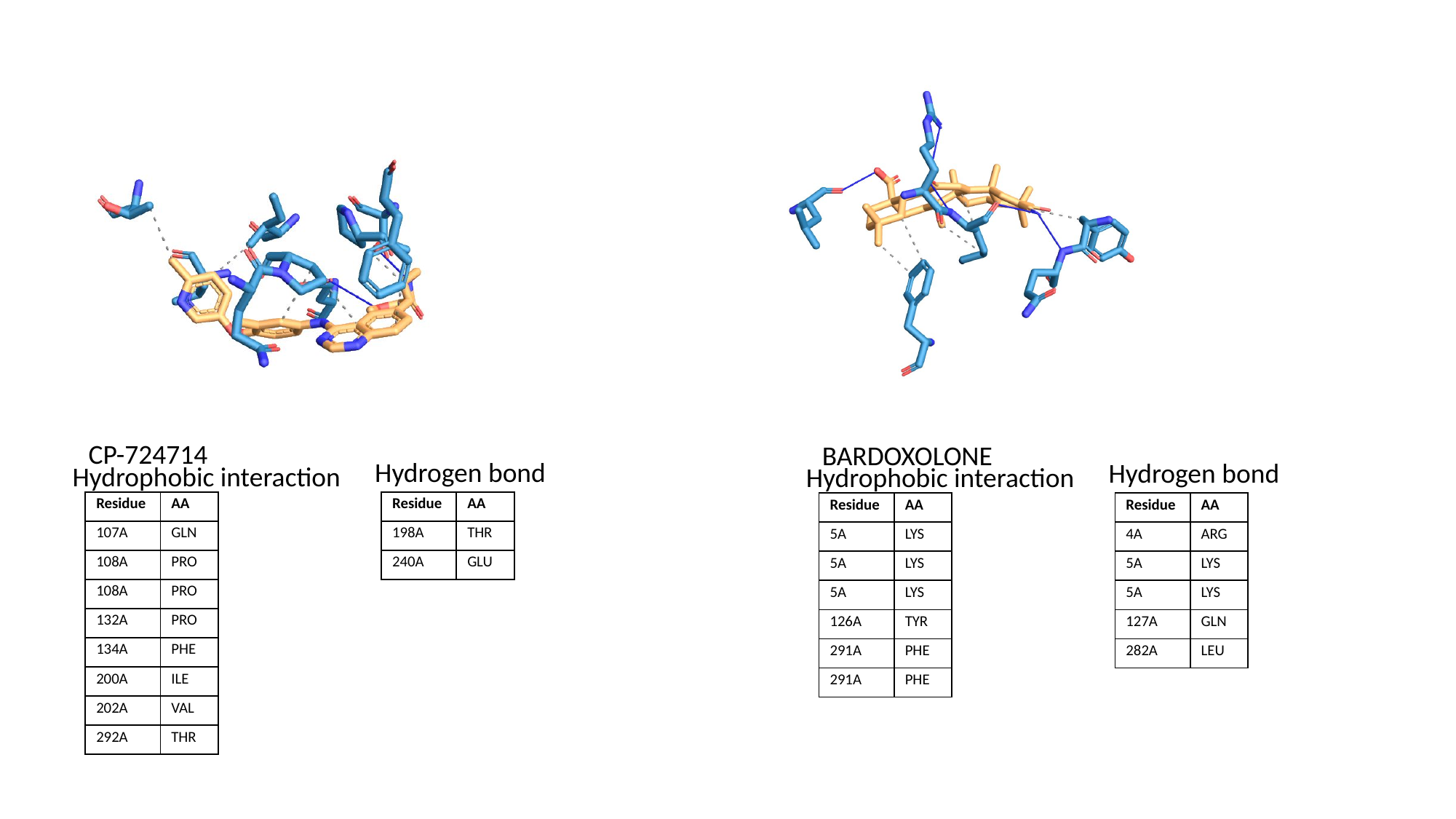

CP-724714
BARDOXOLONE
Hydrogen bond
Hydrogen bond
Hydrophobic interaction
Hydrophobic interaction
| Residue | AA |
| --- | --- |
| 107A | GLN |
| 108A | PRO |
| 108A | PRO |
| 132A | PRO |
| 134A | PHE |
| 200A | ILE |
| 202A | VAL |
| 292A | THR |
| Residue | AA |
| --- | --- |
| 198A | THR |
| 240A | GLU |
| Residue | AA |
| --- | --- |
| 5A | LYS |
| 5A | LYS |
| 5A | LYS |
| 126A | TYR |
| 291A | PHE |
| 291A | PHE |
| Residue | AA |
| --- | --- |
| 4A | ARG |
| 5A | LYS |
| 5A | LYS |
| 127A | GLN |
| 282A | LEU |
